# Comparison of physico-mechanical properties of 3D-printed resins used as permanent indirect restoration materials

**DOI:** 10.2340/biid.v13.46113

**Published:** 2026-05-20

**Authors:** Merve Arıkan, Serdar Akarsu

**Affiliations:** Ordu University, Faculty of Dentistry, Department of Restorative Dentistry, Ordu, Turkey

**Keywords:** 3D printing, dental resin, CAD/CAM, microhardness, surface roughness, flexural strength

## Abstract

**Objective:**

To compare the physico-mechanical properties (microhardness, surface roughness, and flexural strength) of permanent 3D-printed dental resins with those of a resin nanoceramic computer-aided design and computer-aided manufacturing (CAD/CAM) block and a composite resin.

**Methods:**

Four groups were tested: composite resin (Clearfil Majesty Esthetic), resin nanoceramic CAD/CAM block (Cerasmart), and two 3D-printed resins (Saremco Crowntec, VarseoSmile Crown Plus). Disk- and bar-shaped specimens were prepared according to ISO standards. Microhardness (Vickers test), surface roughness (profilometer), and flexural strength (three-point bending test) were evaluated. Data were analyzed using analysis of variance and Tukey post hoc tests (*p* < 0.05).

**Results:**

Cerasmart showed the highest microhardness (85.53 ± 3.97) and flexural strength (184.45 ± 9.42), while Saremco Crowntec exhibited the lowest microhardness (38.63 ± 2.14). Clearfil Majesty had the highest surface roughness (1.24 ± 0.35). Although the measured flexural strength values of 3D-printed resins exceeded the ISO 4049 minimum threshold, several samples were close to or below the limit.

**Conclusions:**

Within the limitations of this in vitro study, 3D-printed permanent resins exhibited mechanical properties comparable to conventional restorative materials in certain parameters, although inferior hardness values were observed compared to CAD/CAM blocks. Nevertheless, the present findings should be interpreted with caution, and further long-term clinical investigations are necessary before translating these results into clinical practice.

## Introduction

Resin-based restorative materials used for definitive restorations include conventional resin composites, resin-nanoceramic computer-aided design and computer-aided manufacturing (CAD/CAM) blocks, and recently developed printable hybrid resins. Conventional resin composites polymerize chairside and present satisfactory esthetics but may suffer from polymerization shrinkage and limited depth of cure [1]. Resin-nanoceramic CAD/CAM blocks, on the other hand, are industrially polymerized under controlled conditions, resulting in a higher degree of conversion and improved mechanical stability [2]. Additively manufactured permanent resins represent a different category in which polymerization occurs layer-by-layer, leading to anisotropic mechanical behavior depending on printing parameters and post-processing procedures [3].

The development of computer-aided applications in dentistry has given rise to the concept of digital dentistry [2]. Treatment methods have evolved from conventional methods to subtractive or additive manufacturing techniques. The digitalization of dental treatments, compared to traditional methods, has shortened working and production times, reduced the number of appointments, and enabled the fabrication of more precise and patient-specific restorations [4, 5]. In the digital dentistry workflow, the final product is fabricated through computer-aided applications using two different manufacturing techniques: subtractive manufacturing (CAD/CAM) and additive manufacturing. Subtractive manufacturing is a method in which the final product is carved from a material block through milling, guided by a computer numerical control (CNC) device [6]. Although CAD/CAM technology has advanced the indirect treatment process, it also presents certain disadvantages, including the complexity of material production, laboratory workflow requirements, excessive waste material, high costs, and prolonged chair time [7]. In addition, the milling process is time-consuming, and its accuracy depends on the geometry of the milling bur.

On the other hand, the fundamental principle of additive manufacturing is the layer-by-layer consolidation of a monomeric material through a computer-controlled energy source, which enables the production of complex three-dimensional structures. This technology allows digital 3D models to be rapidly transformed into physical objects. The process begins with the generation of a digital file in Standard Tessellation Language (STL) format, followed by 3D printing through the joining, binding, or polymerization of small volumetric elements [8]. Additive manufacturing produces only the required parts and allows the reuse of the polymer resin employed in printing, thereby preventing unnecessary material consumption. While subtractive manufacturing is associated with material waste, the post-processing cleaning process performed with IPA (isopropyl alcohol) constitutes a large portion of the volatile chemical emissions in the environment. This is a matter of concern in terms of environmental and occupational exposure [9].

Additive manufacturing also introduces additional processing steps, including washing, post-curing, finishing, and polishing procedures. These steps may increase chairside or laboratory time and involve solvent consumption during cleaning procedures. However, the ability to produce multiple copies or different models simultaneously can save time. In addition, certain optimization strategies, such as designing hollow models, are among the advantages of this method. Although hollow designs are not appropriate for dental restorations, hollow designs are mostly preferred for model production. Inadequate support placement may compromise dimensional accuracy and mechanical integrity, and therefore, support strategies must be carefully balanced rather than minimized. Consequently, the mechanical performance of printed restorations depends not only on material composition but also on printing parameters and build strategy [10, 11]. An important advantage of three-dimensional printing is its capability to fabricate a wide range of materials, including composites, polymers, metals, and alloys [12]. These features make additive manufacturing a highly cost-effective process [13]. Considering all these factors, in recent years, three-dimensional (3D) printing has been proposed as an additive manufacturing approach for indirect restorations [14].

There are several different 3D printing techniques used in the field of dentistry, among which stereolithography (SLA) and digital light processing (DLP) are the most commonly employed methods [15, 16]. While SLA technology generates each layer by tracing points with a laser, DLP printers project the entire layer simultaneously onto the platform with the aid of a digital projector [17]. Being an economically advantageous process has made the integration of resins compatible with 3D printing systems into dentistry a major focus of interest, while the use of these innovative technologies has also enabled the development of novel polymers for biomedical applications [6]. Besides SLA and DLP technologies, masked stereolithography (MSLA-LCD) printing has recently become widely used due to its high resolution and reduced cost [18]. In this system, a monochromatic LCD panel masks ultraviolet light to polymerize an entire layer simultaneously. Compared with laser-based systems, MSLA printers may produce different polymerization kinetics and degrees of conversion, which can influence mechanical properties [19].

Specific parameters such as functional and esthetic properties, marginal adaptation, bond strength, mechanical durability, and resistance to thermal stress are of critical importance for the optimal clinical performance and long-term success of restorations [6]. To achieve satisfactory clinical outcomes, indirect restorations must comply with biological, biomechanical, and esthetic principles. In medium- and long-term clinical success, the mechanical properties of the material, such as its capacity to withstand masticatory forces, flexural strength, microhardness, fatigue resistance, and surface roughness, play a crucial role [20]. Previous investigations have indicated that additively manufactured crown materials may present lower flexural strength values than milled resin-based CAD/CAM blocks, despite generally complying with ISO requirements [21]. It has also been demonstrated that production-related variables such as build orientation and post-curing procedures can significantly influence the mechanical behavior of printed polymers [22]. Furthermore, comparative studies evaluating printable resins and conventional resin composites have reported variations in hardness and surface characteristics depending on the fabrication technique [23].

Despite the growing use of printable permanent crown resins, available data remain fragmented. Previous studies often evaluated provisional materials, different testing conditions, or isolated mechanical parameters. As a result, clinicians lack standardized comparative information regarding how permanent printable resins behave relative to established restorative materials under identical laboratory conditions. Therefore, further investigation of the physico-mechanical properties of 3D dental resins – such as flexural strength, microhardness, and bending resistance – is required. Although several studies have investigated the mechanical behavior of additively manufactured dental resins, most of them evaluated either provisional materials, a single printing material, or compared additive manufacturing only with subtractive techniques. Direct comparison of permanent 3D-printed crown materials with both resin composite and resin-nanoceramic CAD/CAM blocks under identical experimental conditions remains limited. Furthermore, the available literature generally focuses on individual mechanical parameters, while the combined evaluation of microhardness, surface roughness, and flexural strength is scarce. Also in previous studies evaluating the mechanical properties of 3D-printed resins, dental printers such as the Formlabs Form 3B (Formlabs Inc., Somerville, MA, USA) and Asiga MAX (Asiga, Sydney, Australia) have predominantly been utilized. However, studies investigating the performance of budget-friendly printers that are capable of providing clinically acceptable accuracy remain limited.

The aim of this study was to compare the physico-mechanical properties of different permanent three-dimensional resin materials, a resin composite, and a resin nanoceramic CAD/CAM block.

The null hypotheses tested were as follows:

1. There is no difference in microhardness among the tested materials.
2. There is no difference in surface roughness among the tested materials.
3. There is no difference in flexural strength among the tested materials.

## Methods

**Figure 1:**
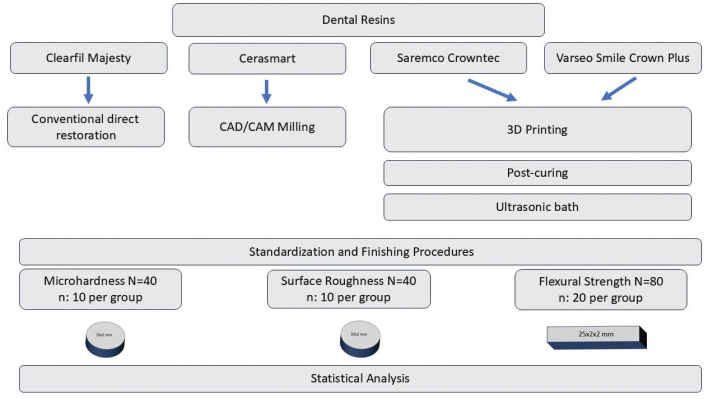
Experimental workflow illustration.

The experimental workflow followed in this in vitro study is illustrated in Figure 1. The materials used in the study consisted of a resin composite (Clearfil Majesty Esthetic, Kuraray Noritake Dental Inc., Japan), a resin nanoceramic CAD/CAM block (Cerasmart, GC Dental Products, Japan), and two different 3D-printed dental resins (Saremco print-Crowntec, Saremco Dental AG, Switzerland; VarseoSmile Crown Plus, BEGO, Bremen, Germany). The properties of the tested resins are presented in Table 1.

**Table 1:** Chemical compositions and manufacturers of restorative materials tested.

Material		Chemical composition	Manufacturer
Clearfil Majesty	CM	Bisphenol A diglycidyl methacrylate (BIS-GMA) Silanized barium glass filler Pre-polymerized organic filler Hydrophobic aromatic dimethacrylate Hydrophobic aliphatic dimethacrylate dl-CamphorquinoneAccelerators, initiators, and pigments	Kuraray Noritake Japan
Cerasmart	CS	71% (w/w): Silica (20 nm) + Barium glass (300 nm) nanoparticlesBis-MEPP, UDMA, DMA, (TEGDMA)	GC Corp. Japan
Saremco Crowntec	SC	BisEMA (50–75% w/w) + ethoxylated bisphenol ester monomers Trimethylbenzoyl diphenyl phosphine oxide (0.1–1%) Silanized glass filler + Pyrogenic silica (total 30–50% w/w)	Saremco, Dental AG Switzerland
VarseoSmile Crown Plus	VSC	Ethoxylated bisphenol-A and 2-methylprop-2-enoic acid esters Silanized dental glass, ~0.7 µm particle size, 30–50% w/w Methyl benzoylformate, diphenyl (2,4,6-trimethylbenzoyl) phosphine oxide	Bego Bremen Germany

The materials used in the study were divided into four groups: Group CM for the resin composite Clearfil Majesty Esthetic, Group CS for the resin nanoceramic CAD/CAM block Cerasmart, Group SC for the 3D-printed dental resin Saremco print-Crowntec, and Group VSC for the 3D-printed dental resin VarseoSmile Crown Plus.

Composite specimens (Clearfil Majesty Esthetic, Kuraray Noritake Dental Inc., Japan) were prepared using a custom-made silicone mold. For the microhardness and surface roughness tests, disk-shaped specimens with a diameter of 10 mm and a thickness of 2 mm were fabricated. For the flexural strength test, bar-shaped specimens measuring 2 × 2 × 25 mm were produced in accordance with ISO 4049 requirements.

During specimen preparation, a transparent Mylar strip and a glass slide were placed on both the upper and lower surfaces of the mold to obtain standardized flat surfaces and to reduce the formation of the oxygen-inhibited layer. Light polymerization was performed using an LED curing unit (3M Elipar Deep Cure, Germany), with an irradiance of approximately 1,200 mW/cm², which was verified prior to use. Disk-shaped specimens were light-cured for 20 s from the top surface. For bar-shaped specimens, polymerization was carried out to ensure complete curing across the entire length of the specimen; given that the tip of the light-curing unit has a diameter of 10 mm, each surface was carefully polymerized in three overlapping sections for 20 s, and after removal from the mold the opposite surface was polymerized in the same manner.

Two types of 3D-printed resins (Saremco print-Crowntec, Saremco Dental AG, Switzerland; VarseoSmile Crown Plus, BEGO, Bremen, Germany) were used to evaluate their physico-mechanical properties. For the flexural strength test, bar-shaped specimens (2 × 2 × 25 mm) were designed in accordance with ISO 4049 standards using the Tinkercad online design software. For the microhardness and surface roughness tests, disk-shaped specimens with a diameter of 10 mm and a thickness of 2 mm were designed.

The designs were exported in stereolithography file format (.STL). Slicing of the designs was performed using Chitubox 1.8 software, and the specimens were printed with a Phrozen Sonic Mini 8K (Phrozen Technology, Hsinchu, Taiwan) printer.

All 3D-printed specimens were fabricated with a 0-degree build orientation, with the long axis of the bar-shaped specimens positioned parallel to the build platform.

Support structures were automatically generated using the slicing software to ensure dimensional stability during printing. The supports were positioned in non-critical areas to minimize surface defects on the designated test surfaces. After printing, all support structures were carefully removed.

For the restorations printed with VarseoSmile Crown Plus (VSC) based on previous studies, the printing parameters were set as follows: 6 bottom layers, 4.5 s exposure time, 40 s bottom exposure time, 8 mm lifting distance, and 60 mm/s lifting speed. The layer thickness was set to 50 µm [13].

For the restorations printed with Saremco print-Crowntec (SC) based on the manufacturer’s instructions, the parameters were adjusted to 6 bottom layers, 4.5 s exposure time, 35 s bottom exposure time, 6 mm lifting distance, and 60 mm/min lifting speed. A layer thickness of 50 µm was selected. After printing, all specimens were removed from the build platform.

For the VSC specimens, the cleaning procedure involved an initial ultrasonic cleaning (Almagest AG, Sofia, Bulgaria) in 96% ethanol for 3 min, followed by immersion in fresh IPA for an additional 2 min. To eliminate residual resin, the specimens were further rinsed with IPA spray and dried using compressed air. For the Saremco print-Crowntec (SC) specimens, the excess material was meticulously removed using a brush soaked in 96% ethanol. Following the cleaning and drying procedures, all specimens were placed in a curing unit (Phrozen Curing Station) at 37°C under a 405 nm light source to complete polymerization. The curing protocol consisted of two cycles of 5 min each.

Sample production from resin nanoceramic CAD/CAM blocks (Cerasmart, GC, Dental Products, Japan) was carried out using a CAD/CAM system. The dimensions and geometry of the specimens were predetermined according to the aims of the study and digitally designed with the Exocad software. The specimen dimensions were modeled three-dimensionally in the software, and the design files were saved in STL format, which is compatible with CAM software. The STL files were then transferred to the CEREC program to enable the milling process. Milling was performed using a five-axis milling machine (Amann Girrbach Ceramill Motion 2, Germany), with the cooling system activated throughout the procedure. Upon completion of milling, the specimens were removed from the device, and any remaining debris and coolant were cleaned from their surfaces using compressed air and distilled water.

All specimen surfaces were finished with silicon carbide papers of 200, 400, and 600 grit to ensure standardization. The central and peripheral dimensions of the specimens (height and width) were measured using a digital caliper with an accuracy of 0.01 mm. Subsequently, the specimens were stored in distilled water at 37°C for 50 ± 2 h.

### Microhardness testing

The microhardness of the specimens was measured using a universal microhardness testing device (FM-700 Microhardness Tester, Future Tech Corp., Japan). The Vickers hardness test was selected for the evaluation. The choice of this method was based on its advantage of employing a calculation formula that is independent of the indenter size and its applicability across a wide range of materials, regardless of their inherent hardness.

During testing, a pyramidal diamond indenter was applied to the specimen surface under a predetermined load for a specific duration. For each specimen, measurements were taken at three different points. A load of 25 g was applied for 3 s at each measurement. Care was taken to ensure that the measurement points were sufficiently distant from the specimen edges and from previous indentation marks to avoid edge effects and interference from adjacent impressions.

The mean of the three measurements for each specimen was recorded as its Vickers hardness value (HV). The HV was automatically calculated by the device software using the standard Vickers formula, based on the microscopic measurement of the diagonals:


$$HV=1.8544\frac{F}{d^{2}}$$

**HV:** Vickers hardness value (kgf/mm²)

**F:** Applied load (kgf)

**d:** Average diagonal length of the indentation left by the pyramidal indenter (mm)

### Surface roughness measurement

The surface roughness of the specimens was measured using a contact-type profilometer (Surftest SJ-400, Mitutoyo, Japan). During the measurements, the diamond-tipped scanning probe was moved across the specimen surface at a predetermined speed and applied contact force. This method was selected due to its high precision in detecting the micro-geometric irregularities of the surface.

For each specimen, three separate measurements were performed on the designated test surface in parallel alignment. During the measurements, care was taken to ensure that the probe moved unidirectionally across the surface and that no vibrations or slippage occurred. To avoid edge effects, the measurement lines were positioned at a sufficient distance from the specimen edges.

The mean of the three measurements for each specimen was recorded as the surface roughness value (Ra) of that specimen. The device automatically calculated the Ra value in micrometers (µm), and the results were documented in the experimental record forms.

### Flexural strength testing

The flexural strength of the specimens was evaluated using a universal testing machine (Shimadzu Co., Kyoto, Japan) employing the three-point bending test method. Each specimen was placed on the support apparatus, and the crosshead loading speed and test speed were set to 0.5 mm/min. The flexural strength (σf) was calculated using the following formula:


$$\sigma \text{f}(MPa)=\frac{3F*L}{2b*h^{2}}$$

**F:** Applied force (*N*)

**L:** Distance between supports (mm)

**B:** Specimen width (mm)

**H:** Specimen height (mm)

**D:** Deflection occurring under the applied force F (mm)

### Statistical analysis

Prior to statistical analysis, the normality of data distribution was assessed using the Shapiro–Wilk test, and the homogeneity of variances was evaluated using Levene’s test. After confirming that the assumptions required for parametric analysis were satisfied, differences among groups were analyzed using one-way analysis of variance (ANOVA), followed by Tukey’s post hoc test for multiple comparisons. All statistical analyses were performed using SPSS software (version 26.0; IBM Corp., Armonk, NY, USA). Results were expressed as mean ± standard deviation (SD). A *p*-value of <0.05 was considered statistically significant.

## Results

### Microhardness

The mean, SD, and minimum–maximum values for microhardness are presented in Table 2. The highest HVs were observed in Group CS (85.53 ± 3.97), while the lowest microhardness values were recorded in Group SC (38.63 ± 2.14). A statistically significant difference was found among the groups (*p* < 0.0001). No statistically significant difference was observed between the HVs of Group VSC and Group SC (*p* = 0.299).

**Table 2:** Means ± standard deviation, minimum, and maximum values of microhardness.

Material	*n*	Mean (SD)	Min	Max	*p*
VarseoSmile Crown Plus	10	39.92^A^ (1.41)	37.00	41.50	*p* < 0.001
Saremco Crowntec	10	38.63^A^ (2.14)	35.30	42.10	
Cerasmart	10	85.53^C^ (3.97)	80.10	93.80	
Clearfil Majesty	10	50.14^B^ (2.74)	46.20	52.90	

SD: standard deviation.

In the table, identical letters indicate no statistically significant difference between groups, whereas different letters indicate a statistically significant difference (*p* < 0.05).

### Surface roughness

The mean, SD, and minimum–maximum values for surface roughness are presented in Table 3. The highest roughness value was observed in Group CM (1.24 ± 0.35), while the lowest value was recorded in Group SC (0.80 ± 0.11). The differences among the groups were found to be statistically significant (*p* < 0.0001). However, no statistically significant differences were detected among the roughness values of Group VSC, Group SC, and Group CS (*p* > 0.05).

**Table 3:** Mean ± standard deviation, minimum and maximum values of surface roughness.

Material	*n*	Mean (SD)	Min	Max	*p*
VarseoSmile Crown Plus	10	0.84^A^ (0.10)	0.72	1.01	*p* < 0.0001
Saremco Crowntec	10	0.80^A^ (0.11)	0.63	1.01	
Cerasmart	10	0.81^A^ (0.14)	0.64	1.12	
Clearfil Majesty	10	1.24^B^ (0.35)	0.79	1.83	

SD: standard deviation.

In the table, identical letters indicate no statistically significant difference between groups, whereas different letters indicate a statistically significant difference (*p* < 0.05).

### Flexural strength

The mean, SD, and minimum–maximum values for flexural strength are presented in Table 4. The highest flexural strength was observed in Group CS (184.45 ± 9.42), while the lowest value was recorded in Group CM (86.85 ± 15.71). No statistically significant difference was found between the flexural strength values of Group VSC and Group SC (*p* = 0.308); however, a significant difference was observed between Group CM and the other groups (*p* < 0.0001). Additionally, statistically significant differences were found between Group CS and the other groups (*p* < 0.001).

**Table 4:** Mean ± standard deviation, minimum, and maximum values of flexural strength.

Material	*n*	Mean (SD)	Min	Max	*p*
VarseoSmile Crown Plus	20	104.78^B^ (10.05)	87.39	124.19	*p* < 0.001
Saremco Crowntec	20	100.85^B^ (9.52)	80.24	117.66	
Cerasmart	20	184.45^C^ (9.42)	170.34	199.37	
Clearfil Majesty	20	86.85^A^ (15.71)	67.06	125.77	

SD: standard deviation.

In the table, identical letters indicate no statistically significant difference between groups, whereas different letters indicate a statistically significant difference (*p* < 0.05).

## Discussion

Recent advances in 3D printing have greatly influenced the fabrication of complex dental restorations [8–24]. The most widely used systems in dentistry are stereolithography (SLA) and DLP, which cure photopolymer layers sequentially [15]. With the development of novel printable resins, permanent composite materials have been introduced for restorative applications [25]. The clinical performance of such restorations depends on parameters such as marginal fit and the physico-mechanical properties of the materials [20]. Therefore, microhardness, surface roughness, and flexural strength are key factors. In this study, the microhardness, surface roughness, and flexural strength of two different 3D-printed permanent resins, a resin nanohybrid CAD/CAM block, and a nanohybrid resin composite were evaluated. According to the results of the study, all null hypotheses were rejected.

The specimens in this study were produced using an 8K MSLA printer. Compared with 4K MSLA or some DLP systems, higher resolution may contribute to improved surface detail, which could influence surface roughness measurements. However, mechanical properties are not determined by resolution alone. Factors such as light intensity, polymerization efficiency, and post-curing procedures may also affect the final material properties [26]. Therefore, the results of the present study should be interpreted within the context of the specific printing system used.

Surface microhardness is a critical parameter for the cohesive strength, wear resistance, and biological compatibility of polymer-based dental materials. This property influences not only esthetic outcomes but also plaque accumulation, the development of secondary caries, and the long-term success of restorative materials [27]. The most commonly employed methods for the evaluation of microhardness are the Vickers and Knoop tests; in particular, the Vickers hardness number (VHN) reflects the material’s resistance to plastic deformation [27, 28]. In the literature, the microhardness of nanohybrid CAD/CAM blocks [29], 3D-printed resins [28] and different resin composites [27] has been investigated, and it has generally been reported that 3D printing materials exhibit lower values. These differences are considered to be associated with the compositions of the materials and their respective manufacturing processes [30]. For instance, in 3D-printed composites, the influence of layer thickness on microhardness has been reported; while a thickness of 50 µm improves surface quality, a thickness of 100 µm, despite reducing printing time, may adversely affect the mechanical properties [31, 32]. In addition, several studies have highlighted a positive correlation between filler content and microhardness [28–33]. The findings of our study are consistent with the literature. No significant difference was observed among the microhardness values of the 3D-printed materials, with the lowest value recorded for Saremco Crowntec. The highest hardness was measured in Cerasmart, which showed a statistically significant difference compared to the other materials.

Surface roughness is a critical parameter that directly influences the biological and mechanical performance of dental materials. These irregularities, which may arise from the manufacturing process, wear, or environmental factors, are commonly assessed using the average roughness value (Ra) [20–34]. According to the literature, the critical threshold value for bacterial adhesion and plaque accumulation has been reported to be 0.2 µm [35]. In our study, Clearfil Majesty was found to be significantly rougher compared to the other groups, whereas Cerasmart, Saremco Crowntec, and VarseoSmile exhibited similar values. These findings are consistent with the results reported by previous studies [20–34]. It is well established that 3D printing parameters influence the surface characteristics of the produced materials [36]. Furthermore, an increase in filler content can reduce surface roughness [36]. Some studies have emphasized that 3D-printed materials exhibit higher surface roughness compared to CAD/CAM blocks [21–38]. In our study, the Ra values obtained were higher than the critical threshold of 0.2 µm. This can be attributed to the layer-by-layer effect inherent to 3D printing technology as well as the measurement methods used.

The differences in surface roughness observed in this study may be largely attributed to the filler content, particle size, distribution and the polishing protocols applied [39]. According to the manufacturers, Saremco print CROWNTEC and BEGO VarseoSmile Crown plus contain approximately 30–50 wt% inorganic fillers; thus, the relatively lower filler loading of these 3D-printed resins may contribute to increased surface roughness. In contrast, Cerasmart has been reported to contain around 70 wt% inorganic fillers, and its more compact filler structure may account for both enhanced mechanical performance and the maintenance of surface gloss after polishing. Clearfil Majesty contains silanized barium glass fillers; in comparison, Saremco and VarseoSmile incorporate silanized glass particles, while Cerasmart includes both silica and barium glass particles. These differences in filler composition may have influenced the surface roughness and microhardness. Moreover, different polishing systems (one-step vs. multi-step, discs, burs, or stones) may yield variable Ra values on the same material [40]. Several studies have demonstrated that the smoothest surface of a restoration is achieved when polymerization is performed under a resin Mylar matrix strip [41]. However, the surface obtained with the Mylar strip is rich in the resin’s organic matrix, which may consequently reduce the surface microhardness of the outermost layer [42].

Flexural strength is a fundamental mechanical property that determines the clinical performance of dental materials and is particularly critical in restorations exposed to high occlusal forces. According to the ISO 4049:2019 standard, the minimum acceptable value for resin-based materials is 100 MPa. However, the reported results for 3D-printed resins vary depending on the printing technology, processing parameters, and post-curing protocols [43, 44]. In our study, Saremco and VarseoSmile exhibited significantly higher values compared to Clearfil Majesty. The highest flexural strength was recorded in Cerasmart, which was found to be significantly different from all other groups. In the literature, VarseoSmile has also been reported to show higher values than Vita Enamic [45] or similarly above 100 MPa [38]; however, some studies have indicated that 3D-printed resins may demonstrate lower strength compared to CAD/CAM blocks and ceramics [46]. Overall, the values obtained for Saremco and VarseoSmile were above the minimum threshold specified by ISO 4049 [47]. Although the measured flexural strength values exceeded the ISO 4049 minimum threshold, several samples were close to or below the limit. Therefore, compliance with the standard should not be interpreted as direct evidence of clinical performance.

This study has several limitations. First, the study was conducted under in vitro conditions, which do not fully replicate the complex oral environment, including variations in saliva composition, pH, temperature fluctuations, and masticatory forces. Second, only a limited number of 3D-printed resins were evaluated, which may restrict the generalizability of the findings to other printable restorative materials currently available. In addition, although standardized printing conditions were applied to ensure comparability, several factors that may influence the physico-mechanical properties of 3D-printed materials – such as variations in printing parameters, layer thickness, printing orientation, support structures, and post-curing protocols – were not investigated. Third, surface roughness was assessed using a contact profilometer, which provides limited information regarding the microstructural or subsurface characteristics of the materials. Finally, long-term performance aspects, including aging, wear resistance, and fatigue behavior, were not evaluated. Therefore, further in vivo and long-term studies involving a broader range of materials and printing parameters are required to provide a more comprehensive understanding of the clinical performance of 3D-printed dental resins.

## Conclusion

Within the limitations of this in vitro study, 3D-printed permanent resins exhibited mechanical properties comparable to conventional restorative materials in certain parameters, although inferior HVs were observed compared to CAD/CAM blocks. The results should be interpreted cautiously, and no direct clinical implications can be drawn without long-term clinical investigations.

## Data Availability

The data used to base the conclusions of the work presented here are available from the author to whom correspondence should be addressed on request.
